# Scanning Photoelectrochemical (Cell) Microscopy for *In Situ* Measurements of Photo(electro)catalysis

**DOI:** 10.1021/cbmi.5c00272

**Published:** 2026-02-03

**Authors:** Kyle Morgan, Namodhi Wijerathne, Md Yeasin Pabel, Bo-Lin Chen, Wei David Wei

**Affiliations:** Department of Chemistry and Center for Catalysis, 3463 University of Florida, Gainesville, Florida 32611, United States

**Keywords:** photo(electro)catalysis, reactivity mapping, anisotropy, heterostructure, electrochemistry, scanning photoelectrochemical (cell) microscopy, electrochemical imaging, charge carrier dynamics

## Abstract

Photo­(electro)­catalysis promises energy-efficient and selective generation of chemical products. However, rational catalyst design is constrained by an incomplete understanding of the underlying mechanisms, necessitating *in situ*, surface-sensitive, and spatially localized characterization techniques. Scanning photoelectrochemical (cell) microscopy (SPECM/SPECCM) utilizes ultramicroelectrodes near the catalyst surface to spatially map structure-dependent activity, charge-transfer dynamics, and active site generation. By directly correlating local structure with catalytic behavior, critical insights into the factors governing catalytic activity, stability, and selectivity are gained, guiding the rational design of high-performance photo­(electro)­catalysts. In this review, we first introduce scanning electrochemical techniques followed by a detailed description of common methods of light integration, underscoring their individual strengths and limitations. We then highlight the ways SPECM and SPECCM have been utilized to gain information on structure-dependent activity in anisotropic materials, charge-transfer in heterostructures, and photogenerated active site formation. We conclude with an outlook on potential research directions to further advance the mechanistic insights obtainable through scanning photoelectrochemical techniques.

## Graphical abstract

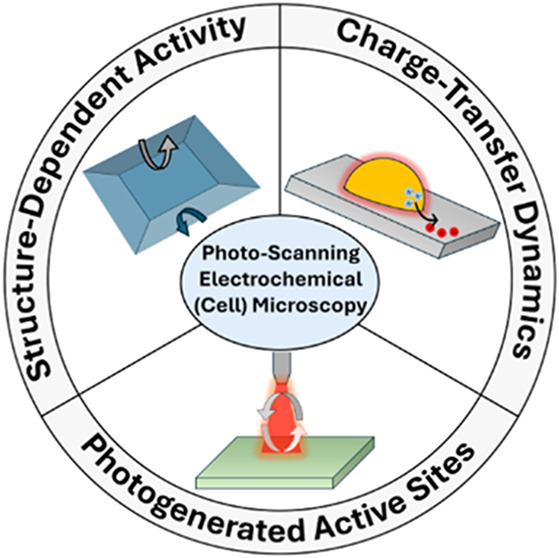

## 1 Introduction

Photo­(electro)­catalysis provides an energetically efficient method of generating value-added chemical products.^1–10^ However, its widespread application is intrinsically limited due to the insufficient stability, activity, or selectivity of most photoactive materials.^1,2,11–13^ To address these fundamental issues, *in situ* characterization is essential.^14–22^ While existing methods have provided some insight into the catalytic mechanism,^14–16,23^ most *in situ* techniques are only capable of measuring an ensemble of particles and/or providing information not confined to the surface.^14,15,17,24,25^ Scanning electrochemical microscopy (SECM)^17,25–32^ and scanning electrochemical cell microscopy (SECCM)^23,33–49^ are emerging techniques that are capable of acquiring such *in situ*, localized, surface-sensitive chemical information.^18,47,50–65^ These techniques consist of ultramicroelectrodes that are brought extremely close (nm−μm) to the surface of the catalytic material through control of piezoelectric motors.^18,29,30,33^ The addition of motors in the x and y planes provides precise spatial resolution to enable scanning of the surface to elucidate site-specific activity, stability, and selectivity.^11,18,20,25,35,60–68^ Most of the experimental studies utilizing these techniques have been performed on electrochemical systems,^21,23,28–31,53,66^ but with the immense potential of photo­(electro)­catalysis, there has been a strong motivation for the integration of light into these methodologies.^9,10,19,28,54–57,67–70^

In photo­(electro)­catalytic systems, it is typically predicted that the dominant active site is relegated to one facet or a type of defect site on the material.^1,28,54,55,71,72^ This emphasizes the need for high-resolution spatial mapping techniques to understand the underlying chemical mechanisms that ultimately contribute to the macroscopically measured activity and selectivity.^16,52,66,73^ Various systems have been studied, including single particles,^9,33,39,57^ anisotropic materials,^43,54,55,74^ and heterostructures^10,19,31,46,63,75–77^ using these photo­(electro)­chemical microscopy techniques. These studies have provided valuable information on charge carrier generation and transport mechanisms as a function of light intensity, wavelength, and applied potential.^9,78,79^ Scanning electrochemical methods are also conducive to high-throughput measurements, allowing for fast and accurate determination of optimized catalysts based on defects, doping, or dominant facets.^39,51,54,80^ Mechanistic insights can also be obtained through active site titration,^20,68,69,81–83^ monitoring reaction intermediates,^21,67,69,84,85^ tracking the charge carrier diffusion,^31,32,44,46,63,70^ and quantifying photothermal heating,^78,86–89^ which directly affects mass transport.

This review will provide a brief overview of SECM, SECCM, and an emerging hybrid SECCM-SECM technique, which will provide a baseline to understand their inherent advantages and disadvantages of gaining chemical information and spatial resolution. This is followed by a description of the common methods of light incorporation into these techniques, with insight into the factors governing the spatial resolution for these configurations. Specific examples are then provided, highlighting the different measurement modalities of these techniques, including substrate-generated tip collection (SG-TC), tunneling SECM, redox competition (RC), surface interrogation SECM (SI-SECM), and carrier-generated tip collection (CG-TC). These examples also show the application of these modalities for elucidating the structure-dependent activity of anisotropic materials, charge transfer in heterostructures, and photogenerated active site formation. Finally, an outline of the current challenges and prospects of this field is provided.

## 2 Instrumentation

The instrumentation and experimental configurations in SECM, SECCM, and SECCM-SECM vary widely depending on the type of experiment and often rely on custom, home-built setups.^17,18,24,27,37,47–49,56,59^ While the integration of light into these systems provides an opportunity to understand the photo­(electro)­chemical processes, it significantly increases the complexity of the system.^42,44,54,68–70^ In this section, we describe the different instrumental setups and experimental methods, including the incorporation of light that are currently being utilized in the field.

### 2.1 Scanning Electrochemical Microscopy (SECM) and Scanning Electrochemical Cell Microscopy (SECCM)

SECM is comprised of either three or four electrodes, where the tip acts as one working electrode (WE 1), substrate acts as a­(n) (optional) second working electrode (WE 2), and the reference and counter electrodes, located far away in the solution (Figure 1A).^17,24,27,30,37^ This instrumental configuration allows for the measurement of generated products or intermediates at the substrate surface.^66,68,90^ A major drawback of SECM is that the substrate is not limited to generating products only where the tip is located, as the entire substrate is under an applied bias.^66,91^ This leads to decreased spatial resolution as the products generated under the tip can diffuse away to the bulk, and the products generated in the bulk can diffuse to the SECM tip.^91,92^ On the other hand, SECCM has the potential for better spatial resolution since the probe itself contains the electrolyte and lacks diffusional flux into or out of the gap.^47–49,63–65,93^ This probe is a nanopipette that forms a drop at the tip, due to capillary action, that, once in contact with the substrate, creates a localized electrochemical cell.^23,31,93,94^ This setup is often only comprised of two or three-electrodes where the substrate acts as the WE, and the other electrode(s) inside the nanopipette act as quasi-reference counter electrode(s) (QRCE) (Figure 1B).^23,94^ While the SECCM configuration offers the potential for higher spatial resolution, it lacks the chemical depth that SECM provides, as the formed products cannot be probed without the second working electrode.^47,59^

**Figure 1:**
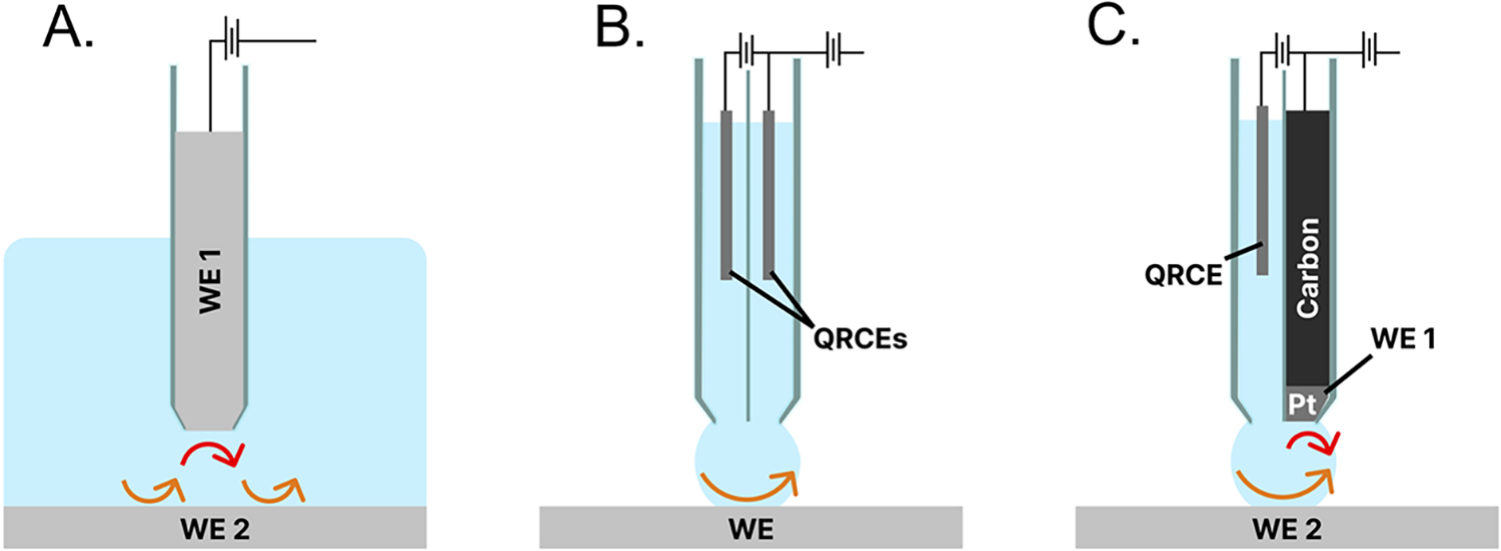
Schematic representation of the instrument setups for (A) SECM, showing a four-electrode configuration consisting of a tip working electrode (WE 1) and a substrate working electrode (WE 2) (the reference and counter electrodes are far away in solution, thus are not depicted here), (B) SECCM, showing a two-barrel, three-electrode configuration with the substrate acting as a WE, and two quasi-reference counter electrodes (QRCEs), and (C) SECCM-SECM in a two-barrel, three-electrode configuration where the additional working electrode allows SECCM to elucidate chemical information.

Recent innovations in this instrumentation have come in the form of the combination of the two techniques, which has been termed SECCM-SECM.^47,58,59^ This was first termed by Paulose Nadappuram et al. in 2015,^47^ where they fabricated a four-barrel pipet with two-barrels being filled with carbon (acting as WEs) and the other two containing QRCEs. While this development showed promise, the difficulty in fabrication limited its widespread adoption by the community. Recently, Ryu and Ren^59^ made an SECCM-SECM probe that contained only a two-barrel pipet, where one side contained a QRCE and the other was filled with carbon with an electrodeposited Pt tip (Figure 1C). Concurrently, Zerdoumi et al. created a similar probe, where the Pt tip was generated by a gas injection method and shaped through focused ion beam milling.^58^ These methods offer a more promising approach to SECCM-SECM, by making the tip fabrication more approachable.

As the focus of this review is specifically on incorporating light into scanning electrochemical techniques, we guide the interested reader to several articles that discuss detailed instrumentation and experimental setup of SECM, SECCM, and SECCM-SECM.^17,23,37,52,66,93,94^

### 2.2 Integration of Light

Integration of light into SECM and SECCM can be challenging due to the complexity of setup and the low current density in photo­(electro)­chemical systems.^64,65^ Depending on the configuration of the light sources, the setups can be categorized as global or local illumination,^68,70,77,78,89^ each with its respective challenges/advantages, which will be discussed here. It is also of note that the practical integration of the light sources does not vary for SECM and SECCM, but they have unique advantages/measurement modalities which will be discussed in Section 3. However, the SECCM-SECM setup has yet to be implemented in photo­(electro)­chemical systems.

#### 2.2.1 Global Illumination

In global illumination, the whole substrate is irradiated either from the front—at an angle— to avoid the tip, or from the back.^77,78,89^ While it is an instrumentally simple and cheap option, the illumination of the whole sample area can lead to various undesired processes. For instance, when the entire substrate performs the photochemical reaction, the products generated in the bulk can diffuse near the tip and can contribute to the signal.^70,73,79^ This leads to decreased resolution and unreliable results,^79^ specifically in the scanning mode of SECM, typically used for determining catalytically active sites and facet-dependent selectivity in photo­(electro)­catalytic reactions.^68^

Additionally, by illuminating the entire substrate, nonstationary effects, including electron or hole accumulation on the surface and subsequent photocorrosion, reconstruction, or phase change, can significantly affect the stability of the electrode.^57,95–98^ Global illumination is also often done with a light source that is at an angle from the front-side of the substrate (Figure 2A). In this configuration, the tip itself partially obstructs the light reaching the tip–substrate gap. Therefore, measuring, or even estimating the light intensity reaching the substrate is difficult. This can be avoided by illuminating the substrate from the back (Figure 2B). However, this configuration is not as common as it complicates the instrumental setup, compared to front side global illumination, and in some cases back illumination has been shown to yield different results than front side illumination for photocatalysis.^99,100^ Local illumination, where the light irradiation is focused, is thus used to mitigate these limitations while improving spatial resolution.

**Figure 2:**
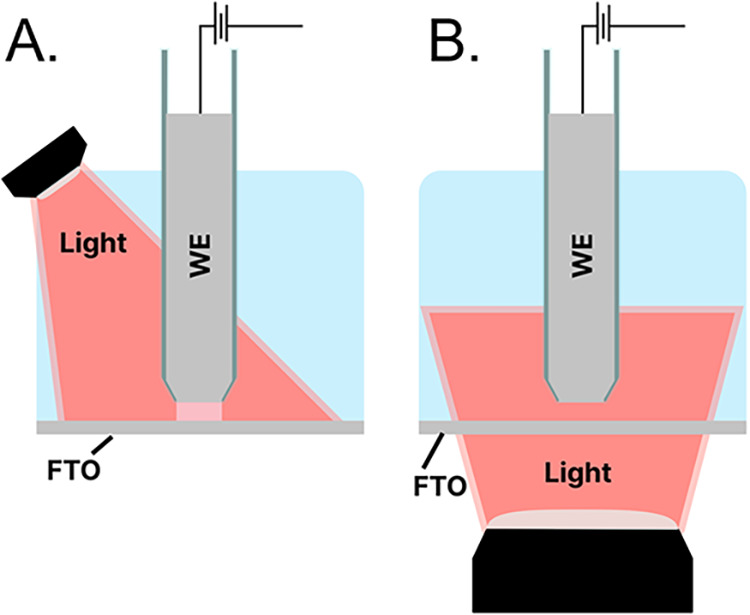
Different global light configurations in SECM: (A) front-side illumination and (B) back illumination. Fluorine-doped tin oxide (FTO) is used as a representative optically transparent electrode substrate.

#### 2.2.2 Local Illumination

Localizing the illumination area has great advantages in minimizing the diffusion of products, reducing photocorrosion, and making the working electrode current specific to the site being measured in SECM.^68,70,79^ Three common configurations—confocal illumination, pinhole-shutter, and through-tip^64,65,68,70,79^ —will be discussed here.

As depicted in Figure 3A, in confocal illumination, the sample is irradiated from the back on an inverted microscope.^19,101^ This instrumentation allows the creation of a registry of particles which otherwise require the use of secondary imaging techniques such as transmission (TEM) or scanning electron microscopes (SEM), with instrument-specific limitations, slowing the overall process.^102^ This method is called optical targeted electrochemical cell microscopy, where the particles are located using hyperspectral imaging to gain insights into their optical properties.^48,103^ When equipped with a diffuse reflectance attachment, the instrument allows monitoring any changes in optical response of the material caused by photo­(electro)­chemical processes,^48^ which is a crucial aspect in photocatalysis. The optical microscopes that are capable of fluorescence measurements are also commercially available with different excitation wavelength filters, allowing for quick and accurate control of wavelength, a particularly attractive feature for plasmonic photocatalysis.

**Figure 3:**
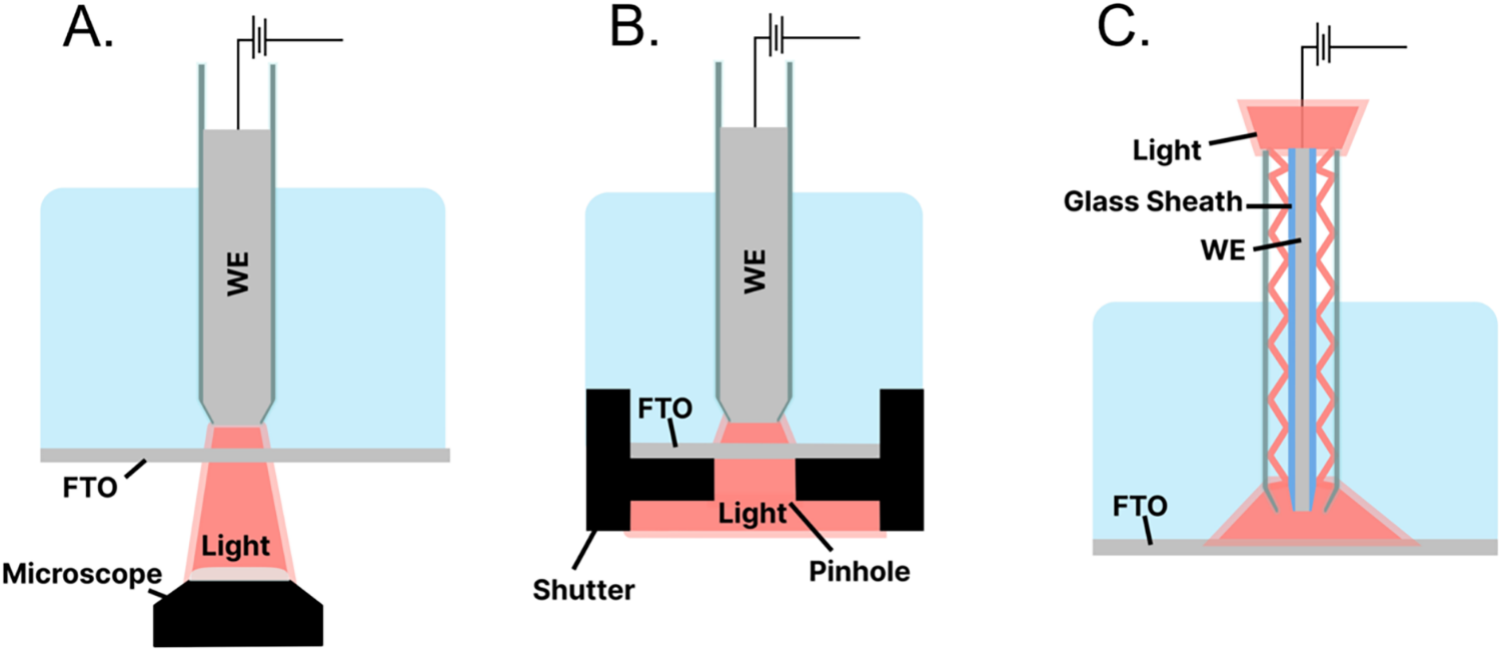
Different local light configurations in SECM: (A) confocal illumination, (B) pinhole-shutter, and (C) through-tip. Fluorine-doped tin oxide (FTO) is used as a representative optically transparent electrode substrate.

A less common approach to local illumination is the use of a pinhole-shutter system (Figure 3B).^68^ This is also a back-illumination method, where a shutter system is placed underneath a thin film of the photoactive material, and the scanning electrochemical tip is brought just above the pinhole of the shutter system.^68^ This allows for tunable illumination area, easy implementation of any light source, and quick and controllable light shut off without turning off the light source itself. The shutter system is also typically time-resolved—a feature used in surface interrogration SECM (SI-SECM)^68^—to perform surface titrations and gain kinetic insights, which will be discussed further in the photo­(electro)­catalytic measurements section.

The third local illumination technique is through-tip illumination.^70,79^ As shown in Figure 3C, the light comes through the scanning electrochemical tip itself. The tip is typically made of borosilicate glass; thus, the light entering at the characteristic acceptance angle, 12.5°, allows the tip to act as an optical fiber.^70^ This local illumination method has the advantage of coming from the front side, which is the more common method of illumination for bulk photo­(electro)­catalysis,^1,5^ and provides measurable light intensity unlike frontside global illumination. While this method seems like it would provide the most localized light source, the illuminated area can be rather large, compared to the tip size.^79^ This is due to the light losing its total internal reflectance upon reaching the region of the glass sheath that was pulled (Figure 3C). The length of this region is typically on the order of micrometers.^79^ Therefore, instead of the tens to hundreds of nanometer resolution of many tips, the light spot is often on the order of micrometers. Nevertheless, through-tip illumination provides a relatively simple way of incorporating local illumination that can be applied to any scanning electrochemical microscopy technique, including SECM and SECCM, as long as a glass sheath is being used.^70^

Even though the spatial resolution in local illumination is much improved compared to that of global illumination,^56,64,70,79^ it is intrinsically limited by either the diffraction limit of the light source, the probe electrode size, or the diffusion length of the photogenerated carriers.^9,31,70,79^ The diffraction limitation can be overcome only when the diffusion length of the carriers is shorter.^17^ For instance, in a multifaceted material, photogenerated carriers diffuse to the lower potential energy surface.^9,104^ For example, in Cu, electrons generated on the (110) facet migrate to the (111) facet, and holes generated on the (111) facet migrate to the (110) facet due to the difference in workfunction (Φ) (e.g., Φ_Cu(111)_ = 4.94 eV and Φ_Cu(110)_ = 4.48 eV below the vacuum level).^104^ If the diffusion length of the carriers is larger than the substrate illumination area, the measured activity would be artificially over/underestimated on a selected facet, leading to unreliable determination of the distinct, facet-dependent catalytic performance.^9^ In a case where the illumination area and the diffusion length of the carrier are smaller than the crystallite domain, the resolution can still be limited if the probe diameter spans multiple domains. Nonetheless, these techniques are powerful in obtaining mechanistic insights, with careful system design.^19,70^

## 3 Photo(electro)catalytic Measurements

Gaining *in situ* information during photo­(electro)­catalytic measurements remains an extremely challenging, yet crucial task for advancing catalyst design.^14–16^ The combination of both precise spatial and chemical insight provided by scanning photoelectrochemical techniques has proven to be invaluable in understanding facet or structure-dependent activity,^9,28,29,43,54,57,60,74^ charge transfer in heterostructures,^10,31,61,63,70,76,77,105,106^ and active site formation.^20–22,56,62,67–69,80–82,84,85,107–109^ This section will discuss different measurement modalities of photoelectrochemical scanning and showcase the unique spatiochemical information they provide in photo­(electro)­catalysis.

### 3.1 Structure-Dependent Activity in Anisotropic Materials

The majority of photo­(electro)­catalytic materials experience some degree of structural heterogeneity either in shape, size, or facet.^9,13,14,28,54,57,110,111^ It is well-known that the optical, electronic, and catalytic properties of different sites of even the same nanocrystal are very distinct.^9,54,71,72,104,110^ Scanning electrochemical techniques offer the unique opportunity to monitor product generation in different areas of anisotropic materials.^9,24,25,28,43,55^

The photogenerated electrons and holes accumulate on different facets in many anisotropic materials,^9,28,54,55^ enabling simultaneous utilization of both carriers, a desirable strategy in photocatalysis to leverage the maximum energy efficiency, such as in overall water splitting (OWS), performing both H_2_ and O_2_ evolution.^9^ In a recent scanning photoelectrochemical microscopy (SPECM) study on a single truncated bipyramidal microcrystal of phosphorus-doped bismuth vanadate (P:BiVO_4_), the photogenerated electrons and holes were accumulated on the dominant {010} and {110} facets, respectively (Figure 4A).^9^ To measure both the O_2_ and H_2_ generation from OWS, a substrate-generated tip collection (SG-TC) mode was used, where the Pt tip electrode was modified with ferrocene (Fc) as a redox mediator.^9^ The modification makes the process an outer-sphere electron transfer, eliminating fouling of the Pt tip, that has been observed on O_2_ reduction and H_2_ oxidation in an inner-sphere electron transfer requiring active participation of Pt during the scan.^9^ This is shown by the stable and consistent photocurrent of the O_2_ reduction of the modified tip in Figure 4B. This technique has been applied with other redox mediators and offers a way of performing many more electrochemical reactions using SECM.^28,43,54,57,60,74^

**Figure 4:**
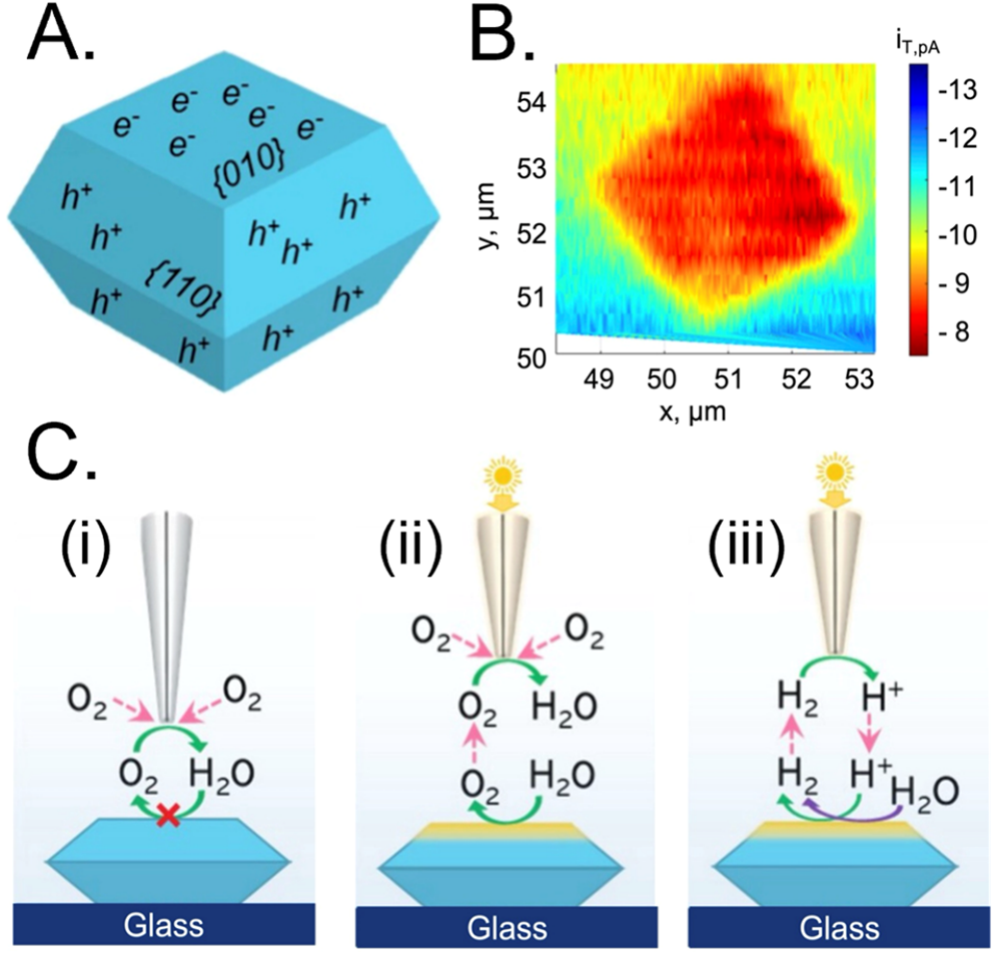
SPECM study on a P:BiVO_4_ microcrystal with (A) facet-dependent carrier accumulation showing (B) O_2_ reduction in positive feedback mode using (C) chopped photocurrent to measure (i) the distance between substrate and tip using O_2_ reduction in a negative feedback mode in the dark to monitor tip–substrate distance, (ii) the O_2_ evolution under light, and (iii) H_2_ evolution under light. Reproduced with permission from ref 9. Copyright 2023 American Chemical Society.

To measure the site-dependent OWS activity of the P:BiVO_4_, a through-tip illuminated (Hg–Xe, ∼0.8 mW) SECM probe was utilized.^9^ Due to geometric differences between the basal {010} and slanted lateral {110} facets, they used dissolved oxygen, and O_2_ reduction-based negative feedback in the dark to control and calibrate the tip–substrate distance (Figure 4C­(i)), and then used chopped illumination to quantify site-dependent activity (Figure 4C­(ii) and (iii)).^9^ The hydrogen evolution was around 6 times greater on the {010} while the oxygen evolution was around 1.5 times higher near the {110} facet.^9^ However, this technique failed to discern the edge (usually ≥ 5 nm) reactivity due to the limited resolution stemming from tip size limitation and the diffusion of species in solution.

A new and developing mode of measuring the local activity with improved resolution is through the use of tunneling mode SECM,^91,92^ which works by placing the tip within a few nanometers (*d* < 3 nm) of a conductive substrate such that electron tunneling between the tip electrode and substrate is facilitated.^91^ For semiconducting substrates, local illumination is essential since photogenerated carriers enable electron tunneling, which is not possible in the dark due to low carrier density.^92^

In tunneling mode SECM, only the tip is biased, whereas the substrate is unbiased (held at open circuit) and typically supported on an insulating material,^91,92^ which differs from traditional scanning tunneling microscopy, where the bias is applied between the tip electrode and substrate.^112^ Tunneling shifts the substrate potential locally, causing the substrate to act as an extension of the tip electrode, enabling Faradaic electron transfer at the substrate–electrolyte interface.^92^ The photo­(electro)­chemical reaction is thus confined to the vicinity of this electrochemically gated region, improving the overall resolution due to the mitigation of the diffusion limitation.^91^

Recently, the Mirkin group used both feedback and tunneling mode SECM to study MoS_2_ nanomaterials.^92^ The mixed-phase MoS_2_ nanosheets, comprised of pseudometallic 1T’ and semiconducting 2H phases, were first studied using the feedback mode.^92^ Ferricyanide (Fe­(CN)_6_^3–^) is spontaneously reduced to ferrocyanide (Fe­(CN)_6_^4–^) on the 1T’ phase, generating a positive feedback loop (Figure 5A­(i)), whereas the 2H phase generated a negative feedback signal due to diffusional hindrance of Fe­(CN)_6_^4–^, caused by the small tip–substrate gap (Figure 5A­(ii)). Interestingly, upon broadband UV–vis illumination in a through-tip configuration, the 2H phase also made a positive feedback loop with the ferricyanide couple (Figure 5A­(iii) and Figure 5B), but as the 1T’ phase was already diffusion-limited, there was no substantial increase in feedback current.

**Figure 5:**
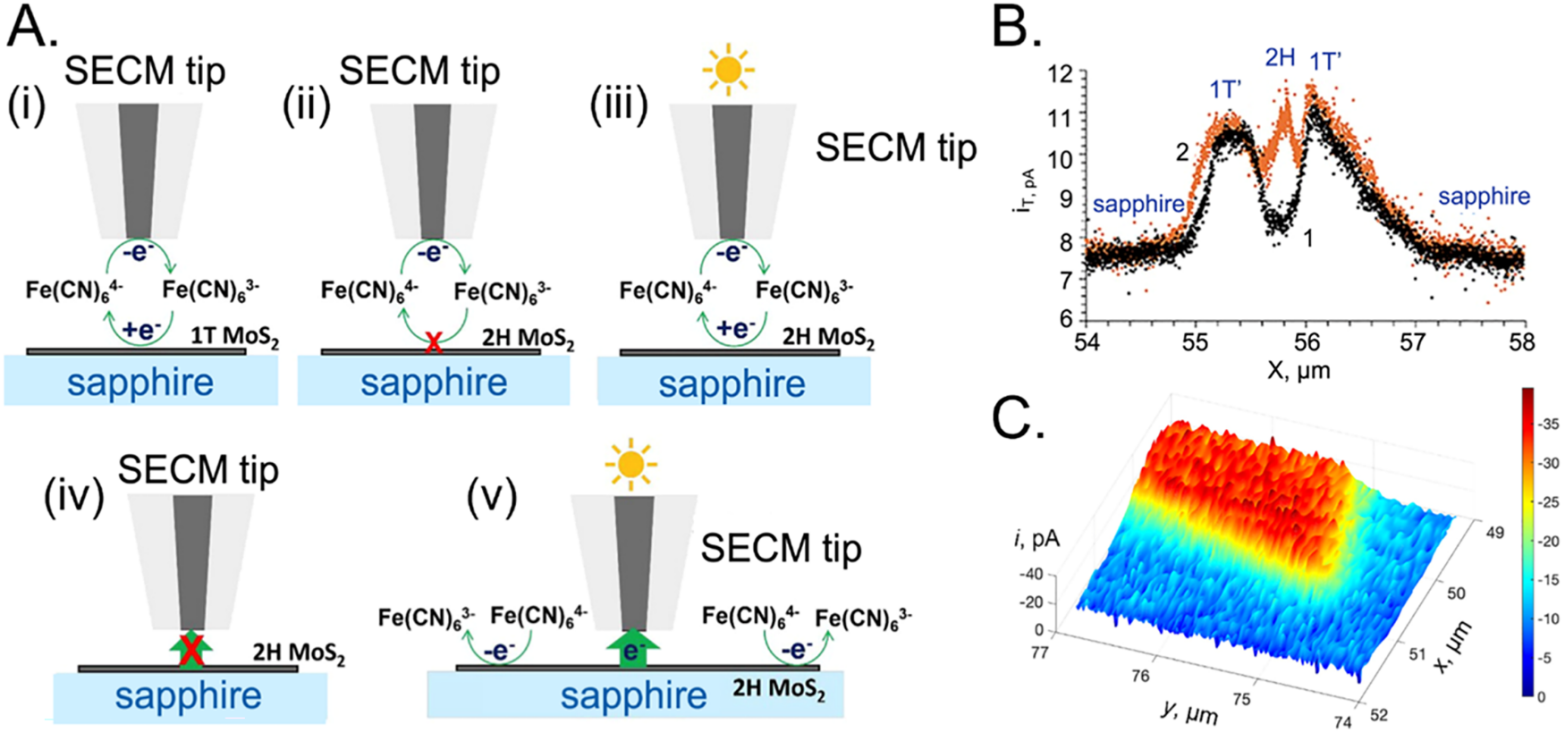
Feedback mode and tunneling mode SECM study on MoS_2_ with (A) the different measurement modalities including: (i) the spontaneous positive feedback loop on the 1T’ phase, (ii) negative feedback on the 2H phase in the dark, (iii) positive feedback loop on the 2H phase during illumination, (iv) lack of tunneling current in the dark on the 2H phase, and (v) tunneling current from the 2H phase to the tip upon illumination, (B) feedback mode current response of the line scan across a MoS_2_ nanosheet in dark (1) and light (2), and (C) tunneling mode current when using protons as the redox mediator on pure 2H phase MoS_2_ when applying a negative bias to the tip to depict the localized HER. Reproduced from ref 92. Copyright 2025 Bo et al. American Chemical Society, Licensed under CC-BY 4.0.

Similarly, when a lateral scan was performed in the tunneling mode across MoS_2_ nanosheet, electron tunnelling was observed only on the 1T’ phase in the dark (Figure 5A­(iv)); however, upon illumination, tunneling was also observed on the 2H phase (Figure 5A­(v), Figure 5B). Interestingly, upon applying a negative bias to the tip, the tunneling current was reversed— as the substrate acted as an extension of the tip, injecting electrons into the 2H phase —allowing the spatial mapping of the H_2_ evolution reaction utilizing protons as the mediator.^92^ Additionally, chemical-vapor deposition-grown 2H phase MoS_2_ nanotriangles acted identically to the 2H phase of the mixed-phase nanosheets, achieving a resolution of about 1–2 nm, offering a promising strategy for increasing the spatial resolution of SECM (Figure 5C). Moreover, when two monolayer nanotriangles were overlapped, they acted as electron trap states, improving H_2_ evolution reaction activity.^92^

SPECCM has also been utilized to elucidate electron transport in anisotropic materials and to measure catalytic activities in photo­(electro)­chemical systems.^28,43,55,57^ For instance, SPECCM was used to examine both the charge transport direction and discern local O_2_ evolution reaction activity at the tips and the sides of the TiO_2_ nanotubes (Figure 6A), without the worry of diffusion, as is necessary in SECM.^55^ The results demonstrated that the photocatalytic activity at the two regions were similar, representative of an orthogonal charge transport, where photogenerated holes transport to the adjacent walls of the tubes (Figure 6B­(i) and (ii)), in contrast to the parallel transport, where the holes transfer to the tips of the nanotubes, in which case, a significant difference in activity can be expected.^55^

**Figure 6:**
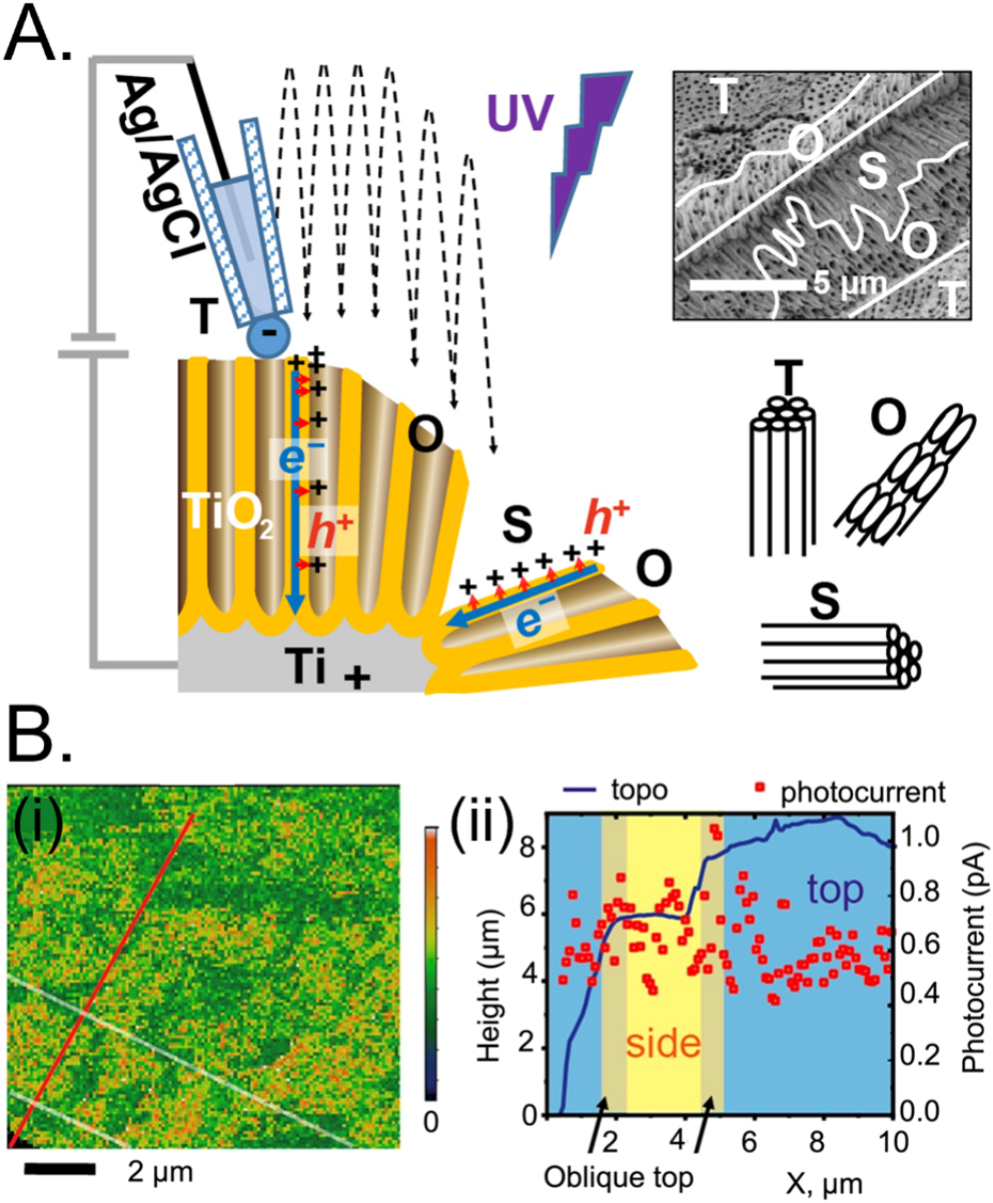
SPECCM study on TiO_2_ nanotubes for investigating charge transport dynamics with (A) depiction of the possible transport model either to the tips (T), oblique tops (O), or sides (S), with the SEM image (inset) (B) (i) photocurrent heat map under 375 nm radiation at 1.5 V vs RHE where the white lines represent the change from O-to-S to O-to-T (starting from bottom left to top right), and (ii) the photocurrent line profile of the red line in (B­(i)) demonstrating, negligible photocurrent change across the different regions. Reproduced from ref 55. Copyright 2022 American Chemical Society.

Utilizing SECCM for single-particle analysis has garnered immense attention recently due to its capability of cataloging an array of different nanoparticles for characterization and locating particles to study their individual activity.^56,58,60,62,64,65^ This is a promising strategy that has found widespread use in electrochemical SECCM for measuring high-throughput activity when the hopping mode is used. Even though such high-throughput studies are desirable in SPECCM, particularly on plasmonic systems where geometry and optical properties are correlated, its practical use is hampered due to very low current. Nonetheless, this direction is expected to be explored due to the strong interest for single-entity photo­(electro)­chemistry.^48,56,64^

These studies highlight the importance of facet and phase-dependent activity and the need for localized techniques to measure anisotropic photo­(electro)­catalysts. The innovation of adding a redox mediator on the tip to prevent it from fouling offers a much more universal strategy for measuring a variety of catalytically driven reactions.^9^ The tunneling mode of SECM is still in its infancy and is expected to make waves in the SECM community, as it will diminish the largest drawback of SECM, its resolution.^91,92^ SECCM has been used on larger particles to understand charge transport and local activities, but it does not typically have the capability to resolve most facet or defect-dependent activities.^54–56,61,64,65^ Increasing spatial resolution of SECCM has the promise to resolve these features with tips having been made down to 20 nm, but low current often hinders practical implementation of these systems for most experimentalists. Single-particle studies using SECCM have been of immense interest in recent years,^28,42,43,46,58,60,63^ and a crossover to photocatalysis is expected to enable high-throughput measurements of single entities.

### 3.2 Charge Transfer in Heterostructures

The use of heterostructures in photocatalytic systems has been a major focus of research over the past five decades.^1,113–117^ Heterostructures allow the spatial separation of the photogenerated electrons and holes, leading to longer carrier lifetimes.^1,113,115^ Consequently, the generation and active regions of each type of carrier are distinct, maximizing the efficiency of the material for photo­(electro)­catalysis.^1,9,113^ The fate of the electrons and holes has been studied using a wide variety of instrumentation, including photoluminescence spectroscopy,^118,119^ transient absorption (pump–probe) spectroscopy,^119,120^ photocurrent measurements,^1,5,113^ Kelvin probe force microscopy,^119,121^ *in situ* electron paramagnetic resonance spectroscopy,^1,5^ etc. However, none of these techniques offer the spatiochemical resolution of scanning electrochemical techniques.^23,51–53^ Such techniques provide the most direct evidence of photogenerated carrier transfer dynamics through the measurement of products, unambiguously elucidating the catalytically active site in heterostructures.^19,31,44,70,77^

Au nanoparticles (Au NPs) on TiO_2_ (Au/TiO_2_) with a Schottky barrier of ∼1.1 eV—a commonly studied heterostructure for catalysis—upon irradiation, separates charge carriers such that the electrons transfer to TiO_2_, leaving the holes near the Au NP interface.^1,7^ In Au/TiO_2_, the plasmonic properties of Au NPs are often leveraged for carrier generation, which is a structure-dependent optical property.^1,7,78,89^ Scanning photoelectrochemical microscopy (SPECM) using global illumination from the back has been employed to study different sizes of Au NPs on TiO_2_ heterostructures with different excitation energies.^19,78,87^

To accomplish this, Au/TiO_2_ heterostructures were constructed by sputtering a Au film through a patterned mask on a TiO_2_ film, which generated Au NPs in a size gradient of 11 nm at the edge to 27 nm in the center, upon thermal treatment (Figure 7A­(i) and (ii), respectively).^19^ This approach allows studying hole accumulation on different sizes of the Au NPs with varying wavelengths of incident light, utilizing ferrocene dimethanol (Fc­(MeOH)_2_) as the redox mediator in SG-TC mode. The instrument was equipped with a spectrophotometer above the sample allowing for measurement of transmitted light as a function of wavelength (Figure 7B,C) and quantification of the external quantum efficiency (EQE) for the hole transfer to the Fc­(MeOH)_2_ (Figure 7D). They concluded that (1) the highest EQE was at the plasmonic peak (2) hole transfer during interband (d–sp), with the smallest Au NPs demonstraes similar EQE at different wavelengths, (3) intraband (sp–sp) transition occurs primarily on the surface whereas the interband transition can also originate in the bulk, and (4) larger particles have a higher EQE than smaller particles under excitation at the plasmon resonance peak (Figure 7D,E).^19^

**Figure 7:**
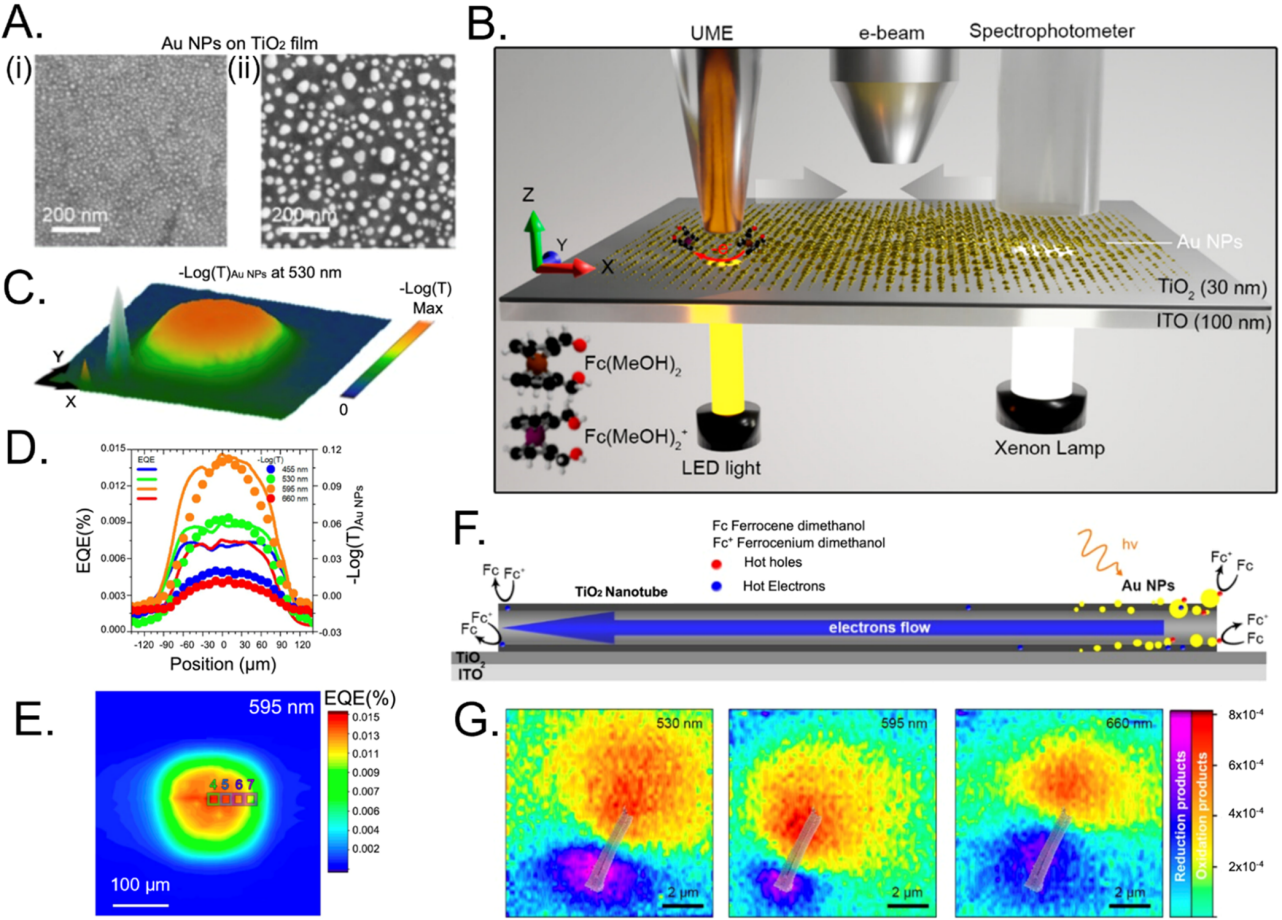
SPECM study on Au/TiO_2_ heterostructures with (A) SEM images depicting the sizes (i) on the edge (11 nm) and (ii) in the center (27 nm), (B) the experimental configuration showing the use of Fc­(MeOH)_2_ as the redox mediator with structural characterization through high-resolution SEM and optical characterization using a spectrophotometer, (C) the absorbance of the patterned masked Au/TiO_2_ at 530 nm, (D) the calculated EQE as a function of wavelength across the patterned mask Au/TiO_2_ at various wavelengths, (E) a photocurrent heat map depicting the EQE at 595 nm across the patterned masked Au/TiO_2_, (F) depiction of the electron transport mechanism across a Au/TiO_2_ nanotube, and (G) photocurrent heat maps showing the spatial separation of electrons and holes due to the reduction and oxidation of the mediator at different wavelengths. Adapted with permission from ref 19. Copyright 2023 American Chemical Society.

Au NPs were then synthesized using the same process as above on TiO_2_ nanotubes to study the electron transfer to individual TiO_2_ nanotubes.^19^ SPECM was utilized to detect areas of hole and electron accumulation through the same Fc­(MeOH)_2_ redox mediator (Figure 7B). The results from the patterned mask method matched well with the hole accumulation results with the nanotubes.^19^ Additionally, this heterostructure with Au NPs spatially confined to the tip of the TiO_2_ nanotube allowed studying the electron transfer by leveraging a redox competition (RC) mode (Figure 7F). In RC mode, the redox mediator is consumed both at the tip and the substrate, enabling quantification of the carriers on the substrate. Here, long-range electron transfer to TiO_2_ from Au NPs was evident due to the decreased tip current caused by the RC, as the hot electrons on TiO_2_ consume Fc­(MeOH)_2_^+^ (Figure 7G). This study confirmed a clear separation of the electrons and holes, on the order of micrometers, and that the maximum EQE for both electron and hole transfer was at the plasmon resonance peak in Au/TiO_2_ heterostrucutres.^19^

The insights gained from this study showcase the use of SPECM for gaining a mechanistic understanding of carrier transport of plasmonic nanostructures on a semiconductor rectifying junction.^19^ While this study is promising, there is far more potential to improve spatial resolution, monitor the different energies of holes on the Au NPs (by using different redox mediators), use a more uniform size distribution of Au NPs, study the effect of a bias potential on the generation/separation, and study anisotropic plasmonic nanomaterials on heterojunctions.

Aside from Schottky junctions, other types of junctions, including semiconductor-semiconductor (typically p–n junctions), have also been widely studied using SPECM.^31,44,63,64,70^ It was recently found that semiconducting MoS_2_ on insulating SiO_2_ could transfer holes from MoS_2_ to SiO_2_.^70^ Using a SPECM with a through-tip illumination with ferrocene (Fc) as a redox mediator, positive feedback was observed on top of a few-layer, 2H phase MoS_2_ triangles (Figure 8A­(i)).^70^ This was attributed to the reduction of ferrocenium (Fc^+^) by photogenerated electrons at MoS_2_, which also displayed an enhanced feedback current toward the perimeter of the MoS_2_ triangle (Figure 8B). Interestingly, when the scan continued to the insulating SiO_2_, a more negative feedback compared to the bulk SiO_2_ was observed in the area adjacent to the MoS_2_ perimeter (laterally 2–3 μm) (Figure 8B,8C).^70^ They correlated this to a RC of the Fc oxidation by the tip electrode and the photogenerated hole accumulation on the SiO_2_ adjacent to the MoS_2_ edges, instead of the normal negative feedback mode due to diffusion hindrance over the bulk SiO_2_ (Figure 8A­(ii)).

**Figure 8:**
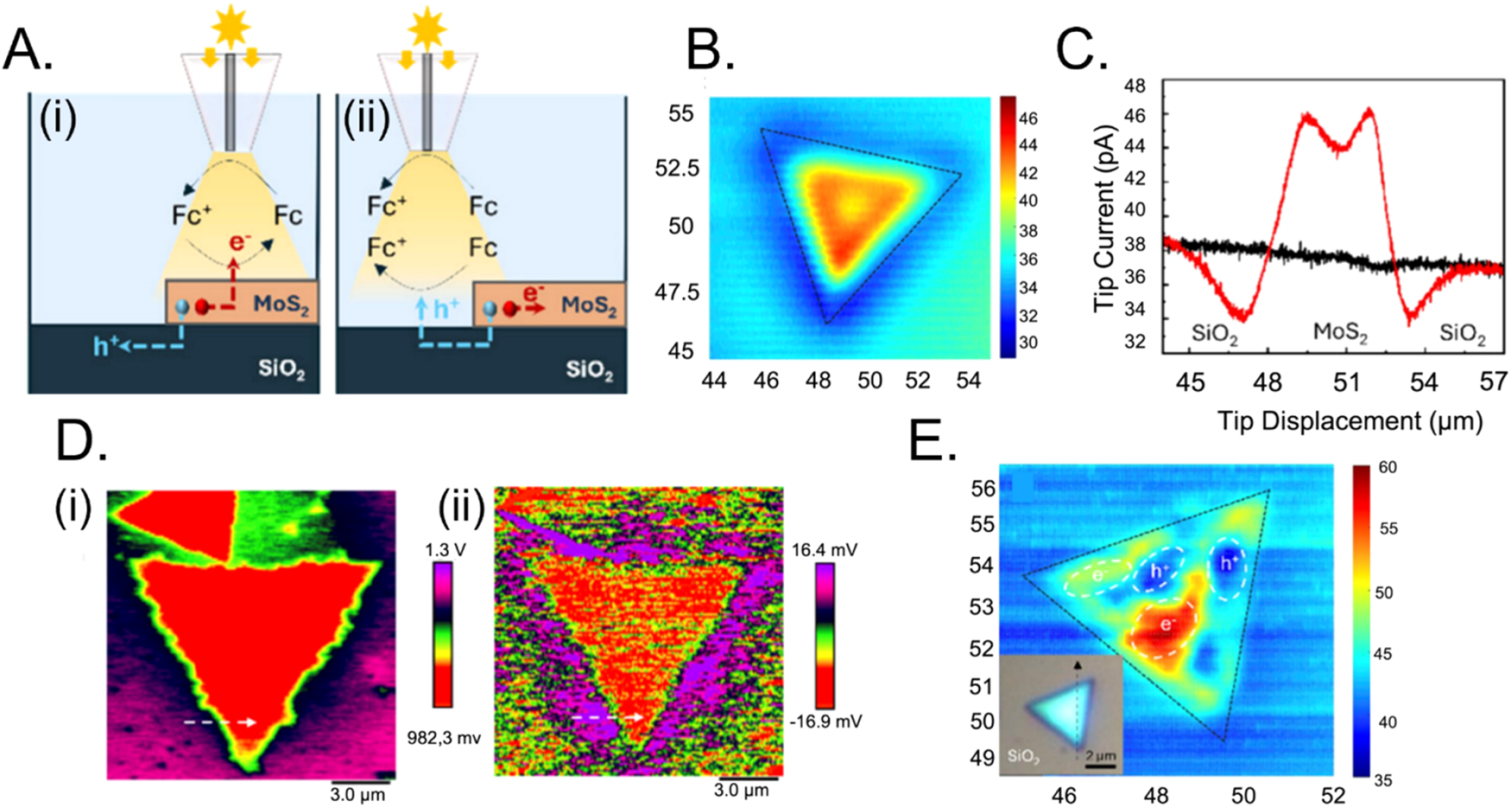
SPECM study on semiconducting MoS_2_ on insulating SiO_2_ with (A) light illumination showing (i) a positive feedback on MoS_2_ and (ii) a RC on SiO_2_ using Fc as the redox mediator, (B) heat map of Fc oxidation at the SECM tip under MoS_2_ illumination with dotted line indicating the edges of MoS_2_, (C) a current line scan across the heterojunction in the dark (black) and under illumination (red), (D) Kelvin force probe microscopy image (i) in the dark-state potential and (ii) surface photovoltage, and (E) a photocurrent heat map of Fc oxidation at the SECM tip for 10-layer MoS_2_. Reproduced from ref 70. Copyright 2025 Wang et al. American Chemical Society, Licensed under CC-BY 4.0.

To further investigate this, spatially resolved surface photovoltage techniques derived from Kelvin probe force microscopy were used.^70^ In the dark, electrons transferred from MoS_2_ to SiO_2_, as evident by the relatively positive and negative surface voltage measurements on the edge of the MoS_2_ and the adjacent SiO_2_, respectively. Upon illumination, the SiO_2_ near the edge of the MoS_2_ became relatively much more positive, consistent with a hole transfer from MoS_2_ to SiO_2_ (Figure 8D­(i) and (ii)).^70^ This observation was correlated to the formation of a space-charge region (SCR) under dark conditions, due to MoS_2_ having a higher Fermi level than SiO_2_, leading to electron transfer to the SiO_2_ defect states. Under light illumination, the SCR drives hole transfer from MoS_2_ to the SiO_2_ defect states along the direction of the built-in electric field. This effect was observed on mono- to 5-layer MoS_2_, while no hole transfer to SiO_2_ was apparent in 10-layer MoS_2_ (Figure 8E). Instead, uneven stacking of the MoS_2_ created defect states which act as carrier accumulation sites, preventing efficient hole transfer to SiO_2_.^70^ Nonetheless, the charge separation between a semiconductor and insulator is quite interesting, as many photocatalytic studies are conducted on insulating SiO_2_, which itself has now been proven using SPECM to be noninert.^70^

The use of scanning electrochemical microscopy has shown great promise in understanding charge carrier dynamics in heterostructures.^19,61,70,77^ While this is true for SPECM, there is only a limited number of studies on heterostructures using SPECCM.^23,45,55,59^ This field is expected to be explored much more as probe sizes get smaller, allowing for better quantification at nanoparticle–substrate interfaces. Tunneling SECM has shown potential in quantifying localized reactivity at extremely high resolutions, as mentioned in Section 3.1, due to the lack of diffusion in traditional SECM modalities. Therefore, it is expected that in photocatalytic studies of heterostructures, the tunneling mode will be utilized for high-resolution spatial mapping.

### 3.3 Insights into Photogenerated Active Site Formation

A central expectation of photocatalysis research is the rational control of the reaction pathways by directing the photogenerated carriers toward the desired reactions while suppressing the parasitic side reactions.^1,121^ In practice, however, macroscopic photocurrent or product measurements rarely provide direct information about the active site.^15,16,22,56,106,109^ SECM and SECCM remedy this limitation, offering direct monitoring of the reaction sites and intermediates, rather than an indirect inference from the averaged signals.^20,56,58,67–69,81–84,90^

In particular, SI-SECM has emerged as a uniquely powerful tool for elucidating reaction mechanisms because it decouples the formation, lifetime, and reactivity of surface oxidizing or reducing equivalents from macroscopic current responses.^20,21,81–84,109^ In the SI-SECM, reactive species are first generated at the substrate by applying a potential pulse and/or illumination. After a controlled delay, the SECM tip is biased to electrochemically generate a redox titrant, which diffuses across the small tip–substrate gap and selectively reacts with the remaining surface-bound intermediates. The resulting tip current indicates the population of the surface intermediates, while its decay with delay time reveals their intrinsic reaction kinetics.^68^

The Bard group demonstrated the use of SI-SPECM for monitoring the generation of surface-bound Fe^4+^ species on a Fe_2_O_3_ electrode using a pinhole-shutter system, as depicted in Figure 3B, for studying the oxygen evolution reaction (OER).^68^ In this study, an ultramicroelectrode tip (radius = 12.5 μm) was brought into close vicinity (3.8 μm) of the Fe_2_O_3_ substrate in a solution of pre-electrolyzed ferrocene methanol (FcMeOH^0^) to generate the oxidized ferrocenium methanol ion (FcMeOH^+^) (Figure 9A­(i)).^68^ Then a light and voltage pulse (ms–s) is applied to the Fe_2_O_3_, generating Fe^4+^ on the surface (Fe_surf_^3+^ + *h* ^+^→ Fe_surf_^4+^) (Figure 9A­(ii)), which is the key intermediate in the OER for the Fe_2_O_3_ substrate. After this pulse, there is a delay time (0–5 s) followed by a reducing potential applied to the tip electrode, reducing FcMeOH^+^. The generated FcMeOH^0^ then acts as a titrant, reducing the Fe^4+^ (Fe_surf_^4+^ + FcMeOH^0^→ Fe_surf_^3+^ + FcMeOH^+^). This generates a positive feedback loop for tip current, and when compared against the dark current (after correction for the collection efficiency and residual oxygen reduction caused by the OER on the Fe_2_O_3_ surface), the density of surface-bound Fe^4+^ sites can be quantified (Figure 9A­(iii) and 9B).

**Figure 9:**
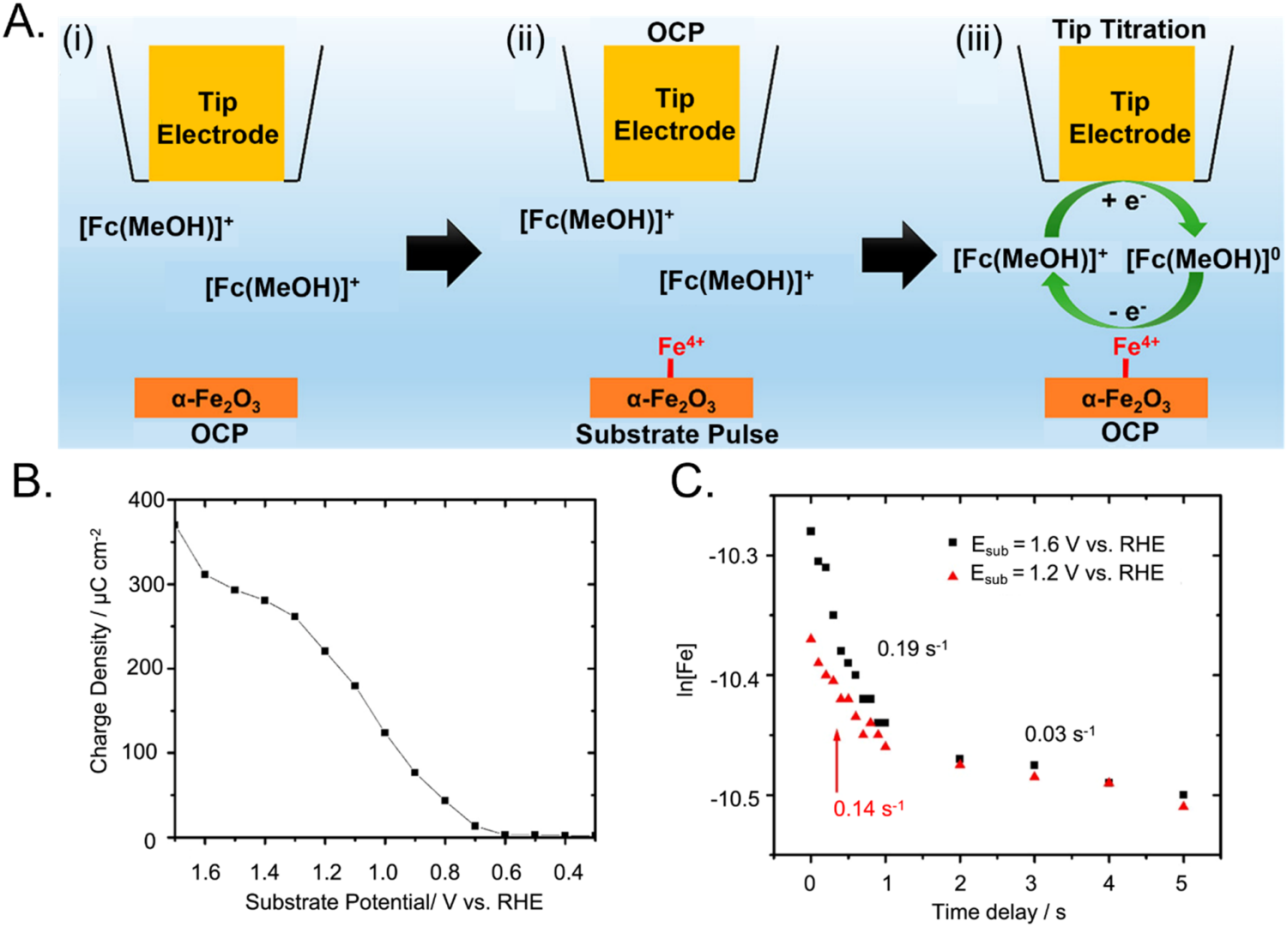
SI-SPECM on a Fe_2_O_3_ photoanode for the OER (A) depicting the experimental configuration where (i) only the Fc­(MeOH)^+^ ion is in solution and only Fe^3+^ is on the surface at open-circuit potential (OCP), (ii) upon illumination and a substrate potential pulse, some amount of Fe^3+^ is oxidized by the photogenerated holes to Fe^4+^, (iii) after a time delay, of not applying a potential or light to the substrate, the redox mediator at the tip is reduced to generate the titrant Fc­(MeOH)^0^ which is then oxidized by the Fe^4+^ state generating a positive feedback current that allows for the quantification of Fe^4+^ states. (B) Plot of the charge density corresponding to the density of surface bound Fe^4+^ states as a function of potential. (C) Results from the time delay titration depicting two pseudo-first order rates at 1.2 and 1.6 V vs RHE. Adapted with permissions from ref 68. Copyright 2018 American Chemical Society.

Additionally, the reaction kinetics of the OER can be obtained by varying the delay time between ending light illumination and applying the tip potential (titrant generation)—feedback current at the tip decreases as the delay time increases—as Fe^4+^ sites are consumed during titration following pseudo-first order kinetics through water oxidation.^68^ For the OER on Fe_2_O_3_, two distinct kinetic regimes were observed corresponding to a “fast” (likely linked to vacancy sites or the defect sites on Fe_2_O_3_) and a “slow” kinetics, yielding two rate constants (0.03 and 0.19 s^–1^) at an applied substrate potential of 1.6 V vs RHE (Figure 9C).^68,90^

Importantly, these insights are inaccessible to conventional voltammetry or steady-state photo­(electro)­current measurements, which reflect charge and mass transport as well as the surface chemistry. Of note, the pinhole-shutter system is necessary in this setup as the tip and substrate are required to be of comparable size to collect all surface Fe^4+^ species generated in the illumination area. This quantitative analysis of surface-active sites is crucial for understanding *in situ* dynamics of photo­(electro)­catalysis, which would be inaccessible without SI-SECM.

Moreover, electrochemical microscopy techniques can be used to decouple surface kinetics from the bulk recombination and the dynamics of excited carriers.^64,65^ SECCM has been utilized to study the carrier dynamics through the carrier-generated-tip collection (CG-TC) modality.^65^ This method, pioneered by the Hill group,^64,65^ utilizes local optical excitation through inverted confocal microscopy, probing the surface at areas of the material relatively far away from the excitation site (Figure 10A).^64^ The use of this method has primarily been utilized on transition metal dichalcogenides for evaluating charge carrier transport, exciton transport, and defect sites.^31,63–65,67,70^

**Figure 10:**
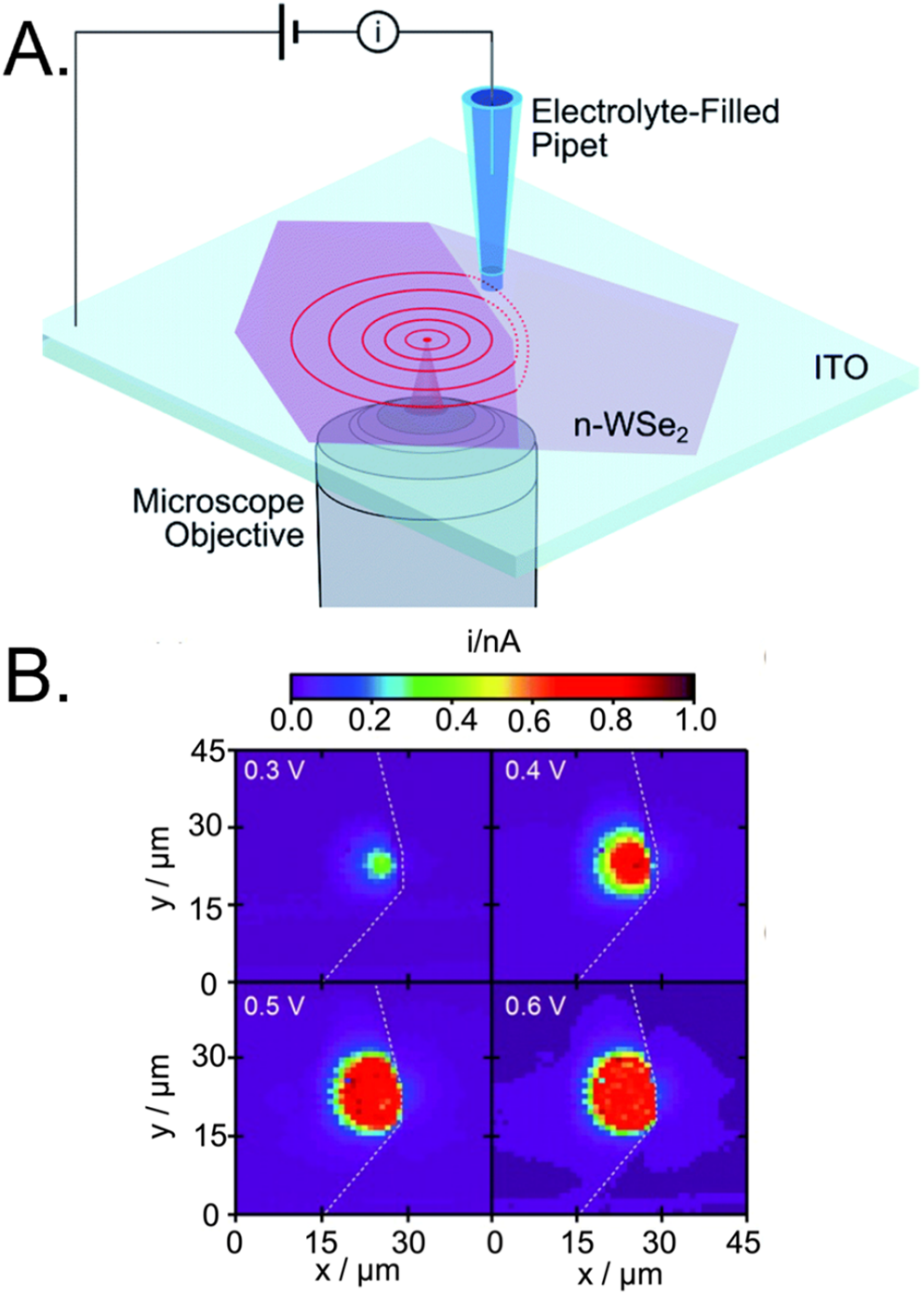
SPECCM in CG-TC mode (A) depicting a schematic illustration of the localized excitation, generating carriers, with the SECCM tip at some distance collecting the carriers on a n-WSe_2_ sheet and (B) the tip measured oxidation current in the WSe_2_ sheet at different potentials with the white dotted lines representing a step edge defect. Reproduced with permission from ref 64. Copyright 2021 Hill and Hill. Royal Society of Chemistry, Licensed under CC-BY 3.0.

Specifically, this has been used on both bulk and monolayer n-WSe_2_ nanosheets.^64^ In bulk n-WSe_2_, hole transport length along the basal planes of a monolayer was found to be 2.8 μm and 5.8 nm in the in-plane (*x*-*y* direction) and out-of-plane (*z* direction), respectively. Additionally, it was observed that step edge defects acted as extremely strong recombination centers, effectively blocking hole transport (Figure 10B). For monolayer n-WSe_2_, the quantum confinement effect causes the exciton binding energy to be much stronger than in bulk n-WSe_2_.^64^ As a result, the diffusion length of the exciton is much longer—more than 20 μm—most likely due to the lack of an interfacial electric field, which can break the exciton binding energy and cause a reaction to occur, generating a current in the CG-TC mode.^65^ It was also observed in this study that to efficiently break the exciton binding energy, a defective step edge site between two overlapping monolayers was required, likely due to surface oxidation of the defective step site generating an oxide layer that induces an internal electric field.

While these studies are not photocatalytic, they do corroborate with other studies in transition metal dichalcogenides, where the step or defect sites act as the active site.^31,56,64,65,67,70^ In bulk p-WSe_2_, it was found that the H_2_ evolution activity was the highest when monolayer step edges were present and that engineering taller step defects resulted in lower H_2_ evoltuionactivity caused by increased recombination.^42,56^ This method provides a valuable means of understanding charge transport in thin films as well as in heterostructures, as it can visualize the true active site under photo­(electro)­chemical conditions.

The use of scanning electrochemical techniques in photo­(electro)­catalysis has given unique insights into the active site generation and understanding carrier dynamics, as highlighted here. These mechanistic insights provide valuable information at a spatiochemical level only conducive to scanning electrochemical techniques.

## 4 Summary and Outlook

The implementation of light into scanning electrochemical techniques is an emerging imaging tool in photo­(electro)­catalysis. Here, we have highlighted the different instrumentation for scanning electrochemical techniques, including SECM, SECCM, and SECCM-SECM, along with the methods in which light has been integrated into these techniques. Using specific examples, we showcase the common uses of scanning electrochemical techniques for photo­(electro)­catalysis, including: the uses of redox mediators (in both SG-TC and RC modes), SI-SECM for quantifying active sites, CG-TC mode for mapping the carrier mobility and dynamics, and the prospects of the tunneling mode SECM for high-resolution imaging.

The techniques applied in most cases are derived strictly from the dark scanning electrochemical experiments, but the implementation of light—though still in its infancy—offers new and exciting opportunities, as different products can be generated, different reaction pathways are created, and a whole suite of techniques applied in photo­(electro)­catalysis can be implemented.

One such technique that has been understudied is the use of intensity-modulated photocurrent spectroscopy (IMPS). This technique utilizes an alternating current waveform directly providing information about recombination rates, trapping, and reaction kinetics. This technique is well-suited for use in SECCM, and to our knowledge has been shown to be viable only once, as the localization of the tip provides much higher resolution than SECM.^49^ IMPS can be used to gain more in-depth information on spatially resolved carrier dynamics. One challenge is the need for a meticulous instrument setup to precisely measure small current fluctuations.

The use of light in the hybrid SECCM-SECM also holds great promise. While photo has been widely used in SECM and moderately implemented in SECCM, the advantage of the spatial resolution of SECCM and chemical information from SECM will further the use of scanning electrochemical techniques. While this field has been limited by the design of the probe tip itself, both the Ren and Schuhmann groups have recently shown easier methods of fabrication.^58,59^

Additionally, the use of more redox probes to measure different reactions such as those involving radical product generation will encourage the photocatalysis community to utilize these methods as a commodity. The generation of radical species is far more common in photocatalytic systems due to the often-high potential energy of electrons and holes. Recently, it was shown that 5,5-dimethyl-1-pyrroline-N-oxide (DMPO) can be used to measure the reactive oxygen species generated from reduction in an SG-TC mode.^200^ This will greatly help in understanding reaction mechanisms and assessing the stability concerns of photocatalysts. Another avenue to implement more redox probes would be to use a series of outer sphere electron transfer redox probes to measure the energy of generated hot carriers on plasmonic nanostructures. This would provide a direct way of determining the steady-state energy and population distributions of hot carriers through a method that does not involve any surface bound species that could alter the absorption or carrier dynamics.

It is of note that SECCM has the potential for high spatial resolution mapping by reducing the size of the probe. However, utilizing smaller probes is challenging due to lower currents. Since typically the current density in photocatalytic systems is lower, the effect is exacerbated in SPECCM. Generally, higher light intensities are used to compensate, by producing higher current densities. However, such high intensities also increase the temperature, causing rapid droplet evaporation. One strategy to increase intensity while mitigating the evaporation is to eliminate the air/water interface. Even though immersing the SECCM tip in an oil has been implemented, its use has been scarce, due to issues with gas phase product transfer across the water/oil interface.^122^ Alternatively, utilizing hydrogel probes has shown promise in SECCM,^37^ which can be extended for SPECCM as long as the hydrogel itself is not photoactive.

Excessive surface wetting is also a major problem in SECCM, especially when using alkaline electrolyte solutions. Incorporating electrolyte additives that are electrochemically inert in the potential window, exhibit no specific interactions with the electrode, and provide droplet cell stabilization at relatively low concentrations, has recently been demonstrated to counteract this issue in alkaline OER.^38,123^ With the use of additives that are not photoactive and thermally stable, this strategy can be established for SPECCM. Therefore, while strategies are being explored, it remains an expanding area of research to implement SPECCM in a traditional SECCM modality, causing this to lag behind SPECM.

From the recent work that has been done using scanning photo­(electro)­chemical microscopies its usefulness is apparent, providing *in situ*, localized, and surface-sensitive information about the chemical products, surface-states, and carrier dynamics on a variety of systems. The implementation of light in scanning electrochemical techniques needs time to reach the mechanistic breakthroughs that SECM and SECCM have achieved. Specifically, the resolution of these techniques is limited due to the spot size, diffusion length, and the diffraction limitations. Even with the gradual improvement in spatial resolution through fabrication of smaller tips and more focused illumination, the field is far from reaching the theoretical limit (diffusion length of the charge carriers). Progress in the field is also hindered due to the complexity of the experimental setups and the need for extremely sensitive, low noise, electrochemical cells, limiting the establishment of new research efforts. Nonetheless, the integration of light is expected to be applied to more systems and ultimately improve our understanding of photo­(electro)­catalytic materials under *in situ* conditions.

## Vocabulary

Photo­(electro)­catalysis: the utilization of light (combined with electricity) as an external stimulus to drive a selective chemical reaction.
Scanning photoelectrochemical microscopy: the use of an ultramicroelectrode probe near the vicinity of an electrode surface to probe *in situ* information about the photo­(electro)­chemical processes occurring on/by the electrode.
Scanning photo­(electro)­chemical cell microscopy: the use of a capillary tube filled with electrolyte and containing a quasi-reference counter electrode to create a droplet electrochemical cell when brought into contact with an electrode surface. This localized electrochemical cell is then used to measure localized photo­(electro)­chemical activities or charge-carrier dynamics.
Anisotropy: the property of a material having different physical characteristics depending on the direction or location at which they are measured.
Spatiochemical: the property of a material having a spatial distribution of chemicals or generated chemical products. Specifically, in heterogeneous catalysis, this refers to the difference in activity/selectivity on different areas of a material.
